# Trends in AIDS Incidence in Individuals Aged 50 Years or Older in the City of Rio de Janeiro, Brazil, 1982–2011: An Age-Period-Cohort Analysis

**DOI:** 10.3390/ijerph110807608

**Published:** 2014-07-29

**Authors:** Paulo Cavalcante Apratto Junior, Mônica Bastos de Lima Barros, Regina Paiva Daumas, Mônica Kramer de Noronha Andrade, Denise Leite Maia Monteiro, Beatriz Rodrigues Lopes Vincent, Valéria Teresa Saraiva Lino, Nádia Cristina Pinheiro Rodrigues

**Affiliations:** 1 Pos-Graduate Program in Medical Sciences, Rio de Janeiro State University, Rio de Janeiro 20550-170, Brazil; E-Mail: aprattoporto@terra.com.br; 2 National School of Public Health Sergio Arouca (ENSP), Oswaldo Cruz Foundation, Rio de Janeiro 21041-210, Brazil; E-Mails: mblbarros@gmail.com (M.B.L.B.); regina.daumas@gmail.com (R.P.D.); monicakra@gmail.com (M.K.N.A.); valeriaslino@gmail.com (V.T.S.L.); 3 Centre for Studies and Research on Ageing, Vital Brazil Institute, Rio de Janeiro 22451-000, Brazil; 4 School of Medical Sciences, Rio de Janeiro State University, Rio de Janeiro 20550-170, Brazil; E-Mails: denimonteiro2@yahoo.com.br (D.L.M.M.); bvincent@lampada.uerj.br (B.R.L.V.)

**Keywords:** acquired immune deficiency syndrome, aging, age effect, period effect, sexually transmitted diseases, epidemiology

## Abstract

*Objective*: The aim of this study was to investigate the effects of three temporal components of AIDS incidence (*i.e.*, age, period and cohort) on individuals aged 50 or older living in Niteroi, Rio de Janeiro (Brazil). *Methods*: Age-specific incidence rates were calculated from 1982–2011. Negative binomial and Poisson models were used to analyze the risk of AIDS by age, period and cohort*. Results*: The risk of AIDS in men was 2.45 times higher than in women, regardless of age and period (*p*-value < 0.001). The incidence of AIDS in individuals older than 69 years was 7-fold lower than in those aged 50–59 years (*p*-value < 0.001). A decreasing trend in AIDS risk was observed from the youngest cohort (≥1940) to the oldest (1910–1919). From 1982 to 2006, we could detect an increasing trend in AIDS risk in the population aged 50 years or older. A peak in rates was detected in the period from 2002–2006. The incidence rates in 2002–2006 were six times higher than those in 1987–1991 (*p*-value < 0.001), independent of age and sex (*p*-value < 0.001). *Conclusions*: An increase of AIDS risk in older people was detected. This group should not be neglected by public health programs.

## 1. Introduction

The increase in life expectancy over the past few decades has resulted in an accelerated population aging process. It is estimated that people older than 59 years currently represent 11% of the global population, and this percentage is expected to increase to 16.5% by 2030. In Brazil, in 2012, estimates indicated that the proportion of the total population older than 59 years is approximately 11.3% (22 million people) and that this value will double over the next 20 years. In developed countries, such as the United Kingdom, Japan, France, Italy and Germany, the percentage of seniors already exceeds 20% of the population. In the United States (USA) this percentage is approximately 16% [1,2].

Changes in the population’s age distribution were accompanied by an epidemiological transition. During the first decade of the AIDS epidemic, few cases were reported in older age groups. However, this number has been steadily increasing [3]. In Brazil, the incidence of AIDS in people aged 50 years or older doubled between 1996 and 2006 [4]. Brazilian national data indicate a gradual increase in incidence rates in older people in the state of Rio de Janeiro until the period 2002–2006; this was followed by a reduction thereafter [5].

Studies outside Brazil also indicate that a significant proportion of the disease affects the older population. In Western Europe (2007) it was reported that 12.9% of newly cases of Human Immunodeficiency Virus (HIV) occurred in people aged 50 years or older [3]. In the United States, approximately a quarter of people with HIV are aged 50 years or older [6,7,8].

In view of the success of antiretroviral therapies, which are able to greatly reduce the likelihood of death in infected people, HIV infection is now considered a chronic condition [9]. In addition, recent studies have shown that treatment is effective for controlling the disease, improving quality of life and decreasing the transmission of the virus [10,11].

Although in Brazil all the population has access to free AIDS treatment regardless of age, the diagnosis of the disease in older people is made late. Usually health professionals do not consider this diagnosis in this age group.

From 1982–2011, reporting of cases in Brazil was only done when the patient with HIV infection met the criteria for AIDS case definition [12]. Even if the person was infected with HIV, the case was only notified and treatment initiated after the establishment of the diagnosis of AIDS. Therefore, there are no official statistics on the incidence of HIV.

The aim of this study was to analyze AIDS incidence from 1982–2011 in individuals fifty years old or older living in Niteroi, Rio de Janeiro by sex, age, period and birth cohort.

## 2. Materials and Methods

This is a population-based study of people aged 50 years or older from Niteroi, Rio de Janeiro. This age group represent 20.6% of the city’s total population. Niteroi is a large urban center neighboring the city of Rio de Janeiro; in 2013, it had 70 public and 280 private health institutions that covered a population of 487,562 [13].

Population data by age, group and sex were obtained from population censuses (1980, 1991, 2000 and 2010), a population count (1996) and inter-census projections (1981–2011). Information on the number of reported AIDS cases by sex, age and year of diagnosis, recorded in the period 1982–2011, were obtained from the Information System for Notifiable Diseases (SINAN), Mortality Information System (SIM) and Control System Laboratory Tests (SISCEL). SINAN data are collected from notification sheets completed in health units [14]. The SIM registry is based on death certificates [15]. SISCEL data come from a network of laboratories that cater to public health.

SINAN is the national surveillance system for AIDS cases in Brazil; however, the Ministry of Health has established a relationship between SINAN and other information systems (*i.e.*, SISCEL and SIM) to reduce reporting delays and the underreporting of AIDS cases. The updated number of SINAN cases based on SISCEL and SIM has been available since 2001. This relationship highlights a significant improvement in reducing the underreporting of AIDS cases.

AIDS cases were defined according to the technical standard of the Ministry of Health definition of AIDS cases in adults for the purposes of epidemiological surveillance [12].

The average incidence of AIDS per 100,000 inhabitants for every five calendar years was calculated based on the weighted average of the number of cases reported during the period divided by the period’s average population, multiplied by 100,000. Average incidence rates by age, birth cohort and period were calculated for men and women using five-year intervals. Negative binomial and Poisson models were used to assess the relationship among the factors (*i.e.*, sex, age, period and cohort) and the incidence of AIDS.

Poisson regression is the most common strategy for modeling count data; however, when models present overdispersion, this technique does not produce reliable estimates [16]. The negative binomial model relaxes the restriction on the dispersion assumptions of the Poisson model. In this study, three different models were used to estimate the rate ratio of AIDS incidence. In model 1, because data did not show significant dispersion, we used Poisson regression. The factors included in this model were sex, age and period. In models 2 and 3, which did not satisfy the Poisson model’s restriction criteria, negative binomial models were used and included, respectively, the variables sex, age and cohort and sex, cohort and period. A logarithmic link function (*i.e.*, logarithm of the population), used as an offset, was added to all models.

Specification of models:

Model 1: * Y*~ *Poisson (µ)*
  *log* (*µ_cases_*) = *α* + *β*_1_ × *sex* + *β*_2_ × *age group* + *β*_3_ × *period* + *log* (*population*)
Model 2: *Y*~ *BN (µ,θ)*
  *log* (*µ_cases_*) = *α* + *β*_1_ × *sex* + *β*_2_ × *age group* + *β*_3_ × *birth cohor* + *log* (*population*)
Model 3: *Y*~ *BN (µ,θ)*
  *log* (*µ_cases_*) = *α* + *β*_1_ × *sex* + *β*_2_ × *birth cohort* + *β*_3_ × *period* + *log* (*population*)

where µ is the average number of expected cases, α is the model intercept and β corresponds to regression coefficients. The age group, birth cohort and period variables were included as dummy variables; the following were used as reference groups: 50–55 years old (*i.e.*, the youngest age group), ≥1940 (*i.e.*, the most recent birth cohort) and 1987–1991 (*i.e.*, the earliest period).

Graphical models were used to present the analyzed results. All analyses were performed with the R-Project software version 3.0.3.

## 3. Results

**Figure 1:**
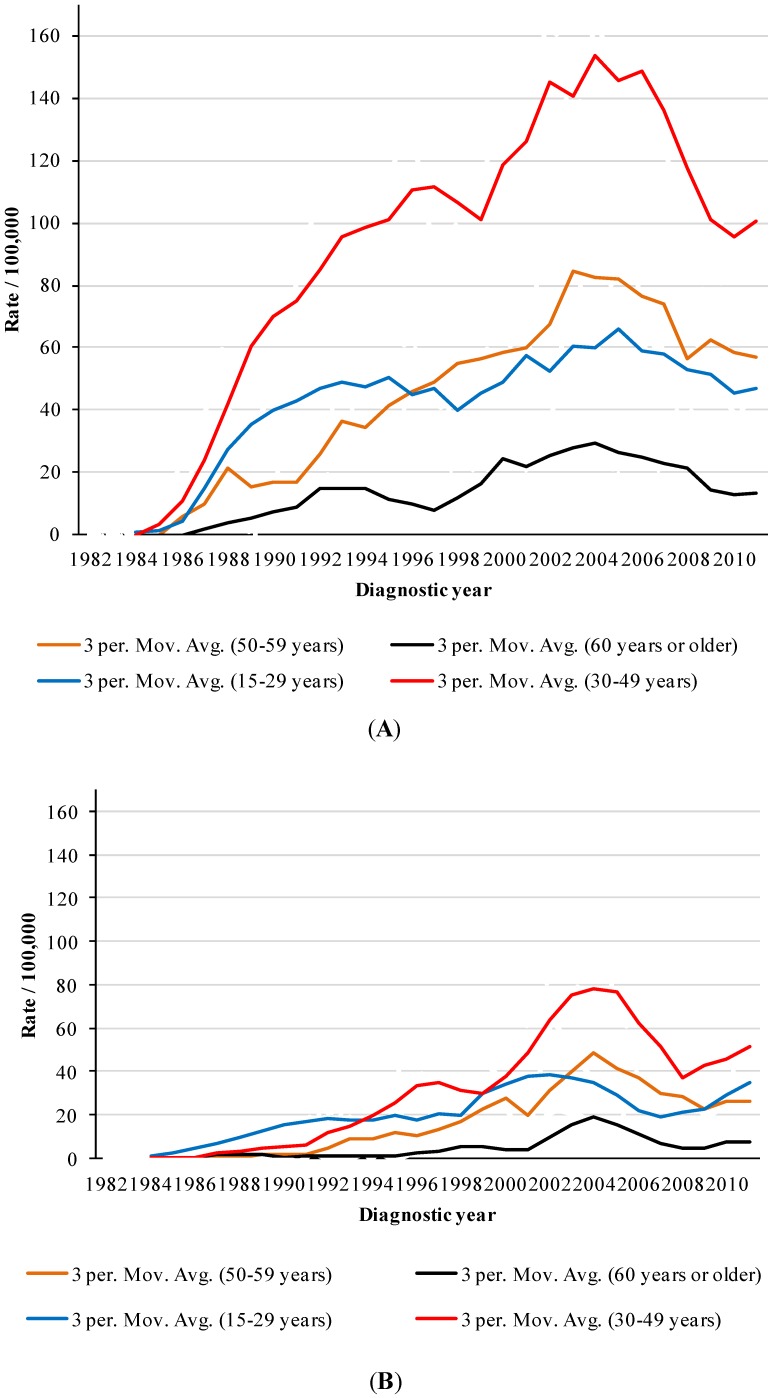
Trends of AIDS incidence per 100,000 inhabitants by period and age group, Niteroi/Rio de Janeiro. (**A**) male; (**B**) female.

From 1982–2011, there were 575 AIDS cases in Niteroi residents aged 50 years or older; 378 in males and 197 in females. The AIDS incidence per 100,000 inhabitants in people aged 50–59 years ranged from zero (1982–1984) to 73.10 (2002), while the incidence in individuals aged 60 or older ranged from zero (1982–1986) to 26.49 (2003) (Figure 1).

Figure 1 shows the trend in AIDS incidence rates by period in different age groups from 1982–2011. The incidence rates represent a moving average (*i.e.*, three year interval). We observed that people older than 59 years presented the lowest rates throughout the period. From 2001–2003, males older than 59 years had rates four times smaller than adults aged 50–59 years, three times smaller than adolescents and young adults (*i.e.*, 15–29 year olds) and approximately seven times smaller than adults aged 30–49 years. Smoothed AIDS rates in males aged 50–59 years exceeded those in adolescents and young adults (*i.e.*, 15–29 year olds) from 1996 to 2010. 

From 2001–2003, the disease rate in women older than 59 years was three times smaller than in adolescents and young adults (*i.e.*, 15–29 year olds) and adults aged 50–59 years. The rate in women older than 59 years was around five times smaller than in adults aged 30–49. Smoothed AIDS rates in females aged 50–59 years exceeded those in adolescents and young adults (*i.e.*, 15–29 year olds) from 2002–2007 (Figure 1).

From 1986–2003, the greatest increase in AIDS rates in men was detected in those aged 60 years or older; the rates were fifteen times greater in 2003 than in 1986. In women, from 1988–2003, the greatest increase was detected in the 50–59 year age group; the rates were 31 times greater in 2003 than in 1988 (Figure 1).

Table 1 shows the AIDS incidence rates per 100,000 inhabitants in individuals aged 50 years or older from 1982–2011. An increasing trend over time, with a peak in the 2002–2006 period, was detected in both sexes. It was observed that the incidence rates in men remained higher than those in women throughout the entire period investigated. While the rate for men almost doubled during the period, in women, the rates became 10 times higher in the same period. The ratio of diagnosed cases in men to women aged 50 years or older has declined gradually over time. The ratio of cases that occurred among men older than 49 years to those in women in the same age group decreased from 3:1 in the 1990s to 2:1 in the 2000s; in the present decade, the ratio is 1:1.

**Table 1:** Descriptive analysis of time series data of the incidence of AIDS (1982–2011) by age-group.

Age Group	Calendar Year *Incidence rate*^1^/*100,000*											
	1982–1986		1987–1991		1992–1996		1997–2001		2002–2006		2007–2011	
	*M* ^2^	*F* ^3^	*M*	*F*	*M*	*F*	*M*	*F*	*M*	*F*	*M*	*F*
50–54	4.3	0.0	29.0	0.0	57.2	10.0	58.6	25.0	76.4	48.7	64.8	28.3
55–59	2.8	0.0	7.5	2.0	25.7	13.2	53.8	14.4	90.2	37.7	36.9	22.1
60–64	0.0	0.0	11.7	2.2	24.2	2.0	33.6	7.9	45.8	28.4	22.2	10.5
65–69	0.0	0.0	4.1	2.9	7.4	2.6	6.3	9.1	36.3	17.4	13.7	7.7
70–74	0.0	0.0	6.1	0.0	11.0	0.0	22.6	0.0	4.0	10.1	21.1	4.5
75–79	0.0	0.0	0.0	0.0	0.0	0.0	15.3	0.0	20.1	3.7	4.8	2.8
≥80	0.0	0.0	12.4	0.0	0.0	0.0	8.3	0.0	7.2	3.2	0.0	0.0

Notes: **^1^** It was used five year average of incidence rates of Niteroi city/Rio de Janeiro; **^2^** Male; **^3^** Female.

Data in Table 1 indicate a decreasing trend in AIDS incidence with age, *i.e.*, the higher the age, the lower the risk of acquiring the disease. In 2002–2006 period, the rate for males in the group aged 80 years or older was approximately 11 times smaller than in the group aged 50–54 years. In women, this reduction was even greater; the rate in the group aged 80 years or older was approximately 16 times lower than in the group aged 50–54 years. In Table 1, we also observe an increasing incidence rate with time period and a peak in the 2002–2006 period for both men and women, followed by a reduction thereafter.

**Figure 2:**
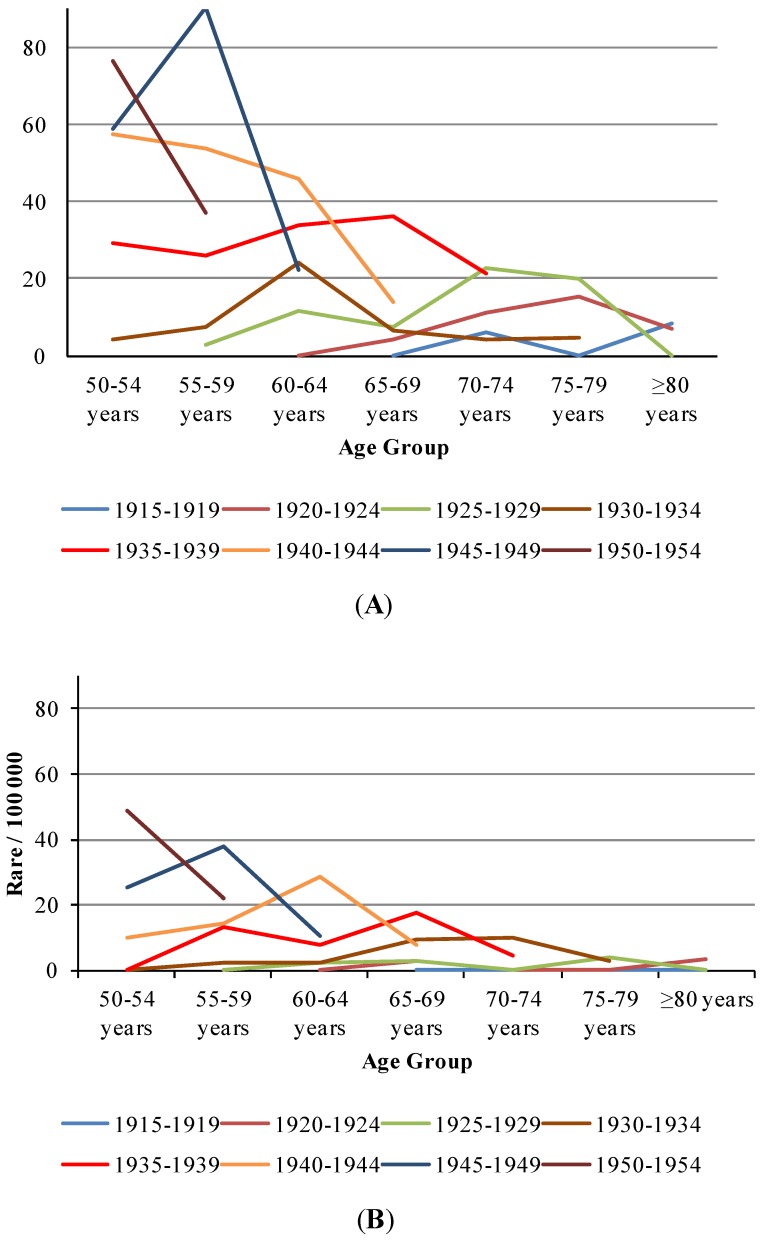
Trends of AIDS incidence **^1^** by period/100,000 in each birth-cohort (age ≥ 50 years) over 1982–2011. (**A**) male; (**B**) female.

Note: ^**1**^ It was used five year averages of incidence rates of Niteroi city/Rio de Janeiro.

An increasing trend with birth cohort can also be detected in Table 1 and Figure 2, *i.e.*, men aged 55–59 born between 1925 and 1929 exhibited the lowest rates of the age group (2.8/100,000), while men of the same age group born between 1930 and 1934, 1935 and 1939, 1940 and 1944 and 1945 and 1949 exhibited incidence rates of 7.5, 25.7, 53.8 and 90.2 per 100,000, respectively. Women born between 1930 and 1934 exhibited a low incidence rate (2/100,000), while those in the same age group born between 1935 and 1939, 1940 and 1944 and 1945 and 1949 exhibited incidence rates of 13.2, 14.4 and 37.7 per 100,000, respectively.

Figure 2 illustrates the age effect on each birth cohort. One can observe a peak from 2002–2006 for all birth cohorts. Men 60–64 years of age born between 1940 and 1944 showed approximately four times greater risk than those born between 1925 and 1929 in the same age group (Figure 2A); in contrast, women aged 60–64 years born between 1940 and 1944 presented a risk that was approximately 12 times greater than those born between 1925 and 1929 in the same age group (Figure 2B).

**Figure 3:**
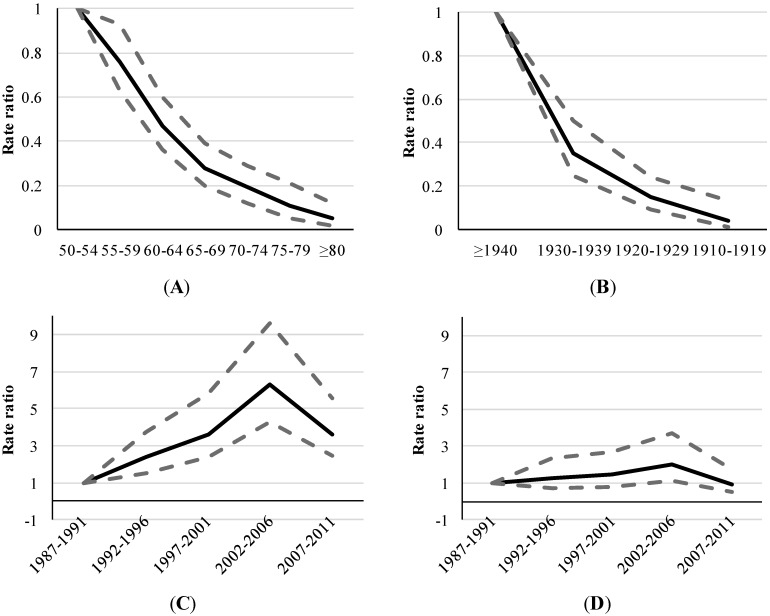
Adjusted rate ratios **^1^** of AIDS by age, period and birth-cohort (age ≥ 50 years), 1987–2011. (**A**) Age effect adjusted by sex and period; (**B**) Birth cohort effect adjusted by sex and period; (**C**) Period effect adjusted by sex and age; (**D**) Period effect adjusted by sex and birth cohort.

Note: **^1^** Three different models were used to estimate the rate ratio of AIDS incidence in Niteroi city/Rio de Janeiro. In the first model, we used Poisson regression and included the factors “sex, age and period.” In the second and third models we used the Negative Binomial models, which included, respectively, the factors “sex, age and birth-cohort” and “sex, birth-cohort and period”.

Table 2 and Figure 3 depict the associations between AIDS and each component of the age-period-cohort triad. The results of multivariate analyses indicate that the risk of developing the disease is 2.45 times greater in men than in women, regardless of age and period (*p*-value < 0.001).

Diagram “A” in Figure 3 illustrates the effect of age on AIDS incidence rates using a model adjusted for sex and period. Individuals aged 70 years or older had a seven times lower risk of disease than those aged 50–59 years (*p*-value < 0.001).

Diagram “B” in Figure 3 shows the birth cohort effect using a model adjusted for sex and period. The rate for the 1910–1919 birth cohort was 20 times lower than that of the ≥1940 cohort.

Finally, diagrams “C” and “D” in Figure 3 represent the period effects from two different models (*i.e.*, “C”, a model adjusted by age and sex and “D”, a model adjusted by cohort and gender). A rate ratio peak is observed in the period from 2002 to 2006 (*p*-value < 0.05); however, the effect seems more marked in diagram “C” than in diagram “D”. The rate of AIDS from 2002 to 2006 was 6.29 times greater than from 1987–1991, regardless of age and sex (*p* < 0.001) (diagram “C”). The rate ratio from 2002 to 2006 was approximately twice as large as it was from 1987 to 1991, regardless of cohort and sex (*p* < 0.05) (diagram “D”). From 2002–2006 to 2007–2011 period, it was observed a decrease of 57% in rate ratio of AIDS (*p* < 0.001) (diagram “C”).

**Table 2:** Age, sex, period, and cohort effects on AIDS incidence **^1^** (age ≥ 50 years) over 1987–2011.

Main Factors	Adjusted Factors	Categories	Coef.	95% CI		RR	95% CI		*p*-value
**Sex**	Age/period	Male/Female	0.9	0.72	1.07	2.45	2.06	2.91	<0.001
**Age-group**	Sex/period	50–54	0	0	0	1	1	1	
		55–59	−0.27	−0.47	−0.07	0.76	0.63	0.93	<0.01
		60–64	−0.76	−1.01	−0.52	0.47	0.36	0.60	<0.001
		65–69	−1.27	−1.62	−0.95	0.28	0.20	0.39	<0.001
		70–74	−1.65	−2.13	−1.23	0.19	0.12	0.29	<0.001
		75–79	−2.21	−3.00	−1.57	0.11	0.05	0.21	<0.001
		≥80	−2.95	−4.13	−2.10	0.05	0.02	0.12	<0.001
**Age-group**	Sex/birth-cohort	50–54	0	0	0	1	1	1	
		55–59	−0.14	−0.56	0.28	0.87	0.57	1.33	<0.51
		60–64	−0.36	−0.83	0.12	0.70	0.44	1.13	<0.15
		65–69	−0.51	−1.09	0.07	0.60	0.34	1.07	<0.10
		70–74	−0.50	−1.22	0.20	0.61	0.30	1.22	<0.15
		75–79	−0.83	−1.82	0.07	0.44	0.16	1.07	<0.10
		≥80	−1.17	−2.55	0.03	0.31	0.08	1.03	<0.13
**Birth-cohort**	Sex/age	≥1940	0	0	0	1	1	1	
		1930–1939	−0.86	−1.26	−0.46	0.42	0.28	0.63	<0.001
		1920–1929	−1.45	−2.11	−0.81	0.24	0.12	0.45	<0.001
		1910–1919	−2.35	−3.91	−1.11	0.09	0.02	0.33	<0.001
**Birth-cohort**	sex/period	≥1940	0	0	0	1	1	1	
		1930–1939	−1.04	−1.39	−0.70	0.35	0.25	0.50	<0.001
		1920–1929	−1.92	−2.43	−1.44	0.15	0.09	0.24	<0.001
		1910–1919	−2.95	−4.38	−1.90	0.05	0.01	0.15	<0.001
**Period**	sex/age	1987–1991	0	0	0	1	1	1	
		1992–1996	0.86	0.43	1.32	2.36	1.53	3.75	<0.001
		1997–2001	1.28	0.88	1.72	3.60	2.40	5.81	<0.001
		2002–2006	1.84	1.46	2.26	6.29	4.29	9.59	<0.001
		2007–2011	1.28	0.89	1.71	3.61	2.44	5.55	<0.001
**Period**	sex/birth-cohort	1987–1991	0	0	0	1	1	1	
		1992–1996	0.22	−0.38	0.82	1.24	0.68	2.27	<0.48
		1997–2001	0.35	−0.23	0.94	1.42	0.79	2.57	<0.24
		2002–2006	0.68	0.11	1.27	1.98	1.12	3.57	<0.05
		2007–2011	−0.09	−0.69	0.52	0.91	0.50	1.68	<0.77

Note: ^**1**^ Three different models were used to estimate the rate ratio of AIDS incidence in Niteroi city/Rio de Janeiro. In the first model, we used Poisson regression and included the factors “sex, age and period.” In the second and third models we used the Negative Binomial models, which included, respectively, the factors “sex, age and birth-cohort” and “sex, birth-cohort and period”.

## 4. Discussion

An age-period-cohort model is a descriptive tool that allows for a comparison of the incidence of AIDS with respect to age, cohort and year of diagnosis and provides an overview of the magnitude of the rates, variation with age and time trends in the rates [17]. This study revealed that age, gender, birth cohort and period were important factors in explaining AIDS incidence in people aged 50 years or older [18,19,20,21].

There is an intrinsic mathematical relationship between cohort, period and age, *i.e.*, the year of birth of the individual represents the difference between calendar time and age [19,20]. Thus, we can say that cohort effects result from the interaction between age and calendar time [21]. Therefore, the changes in AIDS trends by age group over time may be related to changes in risk factors over time that affect each age group differently (e.g., implementation of preventive health policies or changes in the behavior of individuals with regard to contraceptive practices).

As expected, this study detected a strong age effect in both sexes; the rates decrease with age. A study performed in southern Brazil in an older population between 1998 and 2008 found that the highest frequency of AIDS cases were concentrated in the 50–59 year age group (men—75.7%; women—74.7%), followed by the 60–69 year age group (men—18.7%; women—20.7%) and those 70 years or older (men—5.6%; women—4.6%) [22].

Most risk factors related to lifestyle are distributed more homogeneously among individuals belonging to the same generation (*i.e.*, birth cohort) [23]. The cohort effect may result from life experiences of individuals born at a particular point in time, which would influence disease incidence. As a result, the variability of exposures experienced in successive generations could distort the apparent associations between age and disease incidence at a given point in time [21].

Few studies have evaluated the effect of birth cohorts on AIDS rates. Rodrigues (2013) detected similar trends in older people in Rio de Janeiro City, Brazil from 1985–1995 [24]. A study performed on injection drug users aged 15–65 years in Spain detected increasing trends in disease rates in men born between 1950 and 1962 and in women born between 1950 and 1964. For individuals born after these times, the rates were decreasing [23]. In this study, we detected a reduction in AIDS risk in older cohorts, as expected. However, the new generation of seniors already has resources to prolong a high quality of life and increased sexual activity. Moreover, older men are less likely to accept condoms because much of the sexual experience of their generation occurred at a time when AIDS was not yet known, and therefore, condom use was not common. Additionally, the use of condoms to prevent pregnancy is not as necessary in sexual intercourse with women older than 49 years of age.

Period effects associated with incidence rates tend to be most important in diseases for which the cumulative effects of previous exposures are relatively unimportant, as is the case with infectious diseases (e.g., dengue and influenza) [21]. Since the introduction of combined antiretroviral therapy (cART), the life expectancy among people living with HIV has increased significantly. As a result, HIV infection is no longer a disease with rapid progression to death but a disease akin to a chronic condition. Period effects must be analyzed separately from cohort effects (cumulative) because both can change the incidence rates simultaneously. In 1996, cART became available in Brazil. However, only patients who already had a diagnosis of AIDS were treated. Although treatment also reduces the possibility of transmission, the latency period is too long. Probably, the introduction of cART resulted in no significant impact on the incidence in this group until 2006.

The effect of time period on AIDS incidence reported in other studies corroborated our results. Toledo *et al.*, in a study conducted in southeastern Brazil from 1991 to 2006, observed that the cumulative incidence in people aged 50 years or older increased in proportion to the incidence in those aged 20–39 years, peaking in 2005 [25]. Carvalho and Câmara noted a sharp upward trend in AIDS morbidity in the Brazilian population aged 50 years or older throughout the period from 1988–2010 [26]. A study on individuals over 60 years of age in the Brazilian capital identified variations in incidence rates over the period from 1999 to 2009. The highest incidence of AIDS was observed between 2000 and 2005 [27].

We observed higher rates of AIDS incidence in males than in females throughout the observation period, but a greater increase in the rates over time was detected in the female group. This trend in the rates of the different sexes may be related to mechanisms of HIV transmission. From a biological point of view, women are more vulnerable to heterosexual transmission of HIV in unprotected sexual relations than men. This greater vulnerability may be associated with a higher concentration of HIV in semen compared to vaginal secretions [28]. Additionally, the vaginal dryness that occurs at menopause also promotes micro-injuries that increase the risk of transmission of sexually transmitted diseases in women aged 50 year or older [29].

According to a 2010 UNAIDS report, women have accounted for half of the total AIDS cases worldwide [30]. Data from the Brazilian Epidemiological Bulletin corroborate these findings [4]. A study performed in southern Brazil detected that the percentage of cases among women older than 49 years of age rose from 1.6% in 1990 to 7.1% in 2008, representing a 2.5-fold increase over almost 10 years. In men, the percentage increased from 6.5% in 1986 to 9.4% in 2008, which represented a much smaller increase than in women, approximately 15% in 10 years [31].

Sexual life of the older people is still a topic surrounded by prejudice, even among health professionals. This situation delays HIV diagnosis and impairs the treatment of older people living with the disease. The application of HIV tests to confirm the presence of virus in older group was not usually a priority for health professionals. Even with signs and symptoms suggestive of HIV infection, the suspicion of HIV usually is only considered when all other diagnostic possibilities are discarded.

Although our findings represent results obtained from official data, we must consider some limitations of the Brazilian national information system for the interpretation of AIDS incidence trends. Among these limitations, we highlight the changes in the case definition of AIDS through time [12], the deficiency in the coverage of the epidemiologic surveillance system [24], slow data processing, underreporting, the high number of deaths without defined cause and inadequate death certificate completion. In addition to the cases that were not reported, data in this study excluded asymptomatic infected individuals because these data are not yet available in Brazil. The quality of data from the Brazilian information systems is being improved slowly and gradually [32,33].

## 5. Conclusions

The trend of the incidence rates in people aged 60 years or older was approximately proportional to the increase in people aged 15–29 years, while the increase in rates in people aged 50–59 years was proportional to the increase in people aged 30–49 years. Despite recent changes in older cohorts, such as an increase in the AIDS incidence rate, an increase in life expectancy and a longer sex life [34], there is no public prevention policies aimed at this age group.
